# Reconstituting central nervous system niche cues partially restores homeostatic-like features in cultured murine primary microglia

**DOI:** 10.1038/s41598-026-61142-0

**Published:** 2026-07-07

**Authors:** Victoria Ilse, Susan Barendrecht, Julia Hahndorf, Stefanie Geissler, Benjamin Hietel, Daniel-Christoph Wagner, Martin S. Staege, Holger Cynis

**Affiliations:** 1 Department of Drug Design and Target Validation, Fraunhofer Institute for Cell Therapy and Immunology, Halle (Saale), Germany; 2 Present Address: Child Brain Lab, Sophia Children’s Hospital, Erasmus University Medical Center, Rotterdam, The Netherlands; 3 Present Address: Institute for Radiology, Charité – Universitätsmedizin Berlin, Corporate Member of Freie Universität Berlin and Humboldt-Universität zu Berlin, Berlin, Germany; 4 Institute of Pathology, University Medical Center Mainz, Mainz, Germany; 5 Department of Surgical and Conservative Pediatrics and Adolescent Medicine, Faculty of Medicine, Martin Luther University Halle-Wittenberg, Halle (Saale), Germany; 6 Junior Research Group, Immunomodulation in Pathophysiological Processes, Faculty of Medicine, Martin Luther University Halle-Wittenberg, Halle (Saale), Germany

**Keywords:** Primary microglia, Culture shock, Homeostatic gene signatures, CNS niche cytokines (hTGF-β1, hIL-34, hCX3CL1), Cell biology, Immunology, Neuroscience

## Abstract

Microglia rapidly lose their homeostatic phenotype after isolation, limiting the interpretability of in vitro studies. We systematically evaluated culture conditions that reconstitute central nervous system (CNS) niche inputs to better preserve microglial homeostatic features. Neonatal mouse microglia were isolated by fluorescence-activated cell sorting, Percoll gradients, or a shaking protocol and cultured with defined cytokines (TGF-β1, IL-34, CX3CL1), extracellular matrix (collagen IV), and metabolic support (cholesterol, insulin-transferrin-selenium) under serum-free or serum-containing conditions. RT-qPCR revealed rapid downregulation of seven homeostatic transcripts (Tmem119, P2ry12, Cx3cr1, Hexb, Fcrl2, Olfml3, Tgfbr1) within 24 h across isolation methods, with further decline for a subset over 7 days. A cytokine cocktail partially restored homeostatic gene expression, with collagen IV and cholesterol further enhancing selected transcripts. Serum-free conditions favored ramified, surveillant-like microglia, whereas serum promoted amoeboid, activated-like cells, and adding defined factors preserved ramification while improving transcriptional recovery. Transcriptomic analyses demonstrated that optimized conditions shifted global expression profiles toward neonatal ex vivo microglia, upregulated most microglia-specific homeostatic genes, and downregulated inflammatory effectors. These data define a scalable, serum-free, collagen IV-based culture system with defined CNS cues that partially preserves key aspects of microglial homeostatic transcriptional and morphological features compared with conventional serum-containing culture, providing more physiologically relevant conditions for mechanistic and disease-relevant studies.

**Supplementary Information:**

The online version contains supplementary material available at 10.1038/s41598-026-61142-0.

## Introduction

Microglia are the resident immune cells of the central nervous system (CNS) and are essential for maintaining brain homeostasis. They mediate synaptic pruning and the clearance of cellular debris while contributing to the formation of neural circuits and cognitive processes through complement-dependent synapse elimination^1–3^. Critically, microglia adopt a unique homeostatic phenotype in vivo, characterized by highly ramified morphology and expression of signature markers including transmembrane protein 119 (*Tmem119)*, purinergic receptor P2Y12 (*P2ry12)*, and CX3C motif chemokine receptor 1 (*Cx3cr1)*^4,5^. However, this phenotype is rapidly destabilized upon isolation and in vitro culture, due to loss of interaction with other cell types within the brain microenvironment^6^. This leads to profound transcriptional reprogramming of microglia within hours – a phenomenon termed “culture shock”^7,8^. During this transition, homeostatic genes (e.g., *Tmem119*, *P2ry12*) are markedly downregulated, while genes associated with activation, proliferation, and inflammation (e.g., *Apoe*, *Spp1*, *Lyz2*) are upregulated^9^. Serum-containing culture conditions have been shown to further enhance phagocytic activity and to induce transcriptional upregulation of cytokines such as tumor necrosis factor alpha (TNF-α) and interleukin-6 (IL-6)^8^.

This phenotypic drift poses a major translational barrier. In vitro-cultured microglia develop increased heterogeneity and acquire disease-associated activation states not reflective of their physiological in vivo counterparts, thereby limiting the relevance of findings to CNS conditions^10^. The loss of homeostatic signatures in vitro is attributed to the absence of CNS-specific microenvironmental cues. In vivo, microglial quiescence and identity are maintained through dynamic neuron-glia signaling axes, including neuronal fractalkine (CX3CL1), which promotes microglial surveillance and suppresses activation^11,12^. Astrocyte-derived transforming growth factor beta 1 (TGF-β1) is equally indispensable, governing microglial development and homeostatic gene expression^4,13^, while neuron-secreted interleukin-34 (IL-34) supports microglial survival via colony stimulating factor 1 receptor (CSF1R)^14–17^. Recent advances demonstrate that integrating these cues can partially restore physiological phenotypes, as evidenced by induced pluripotent stem cell (iPSC)-derived microglia generated using cytokine cocktails that mimic the CNS niche^10^.

While adult microglia isolated by fluorescence-activated cell sorting (FACS) provide authentic transcriptional profiles, their low yield and technical complexity restrict widespread application. Consequently, primary microglia cultures remain the predominant model despite their compromised physiological fidelity, a gap that critically impedes mechanistic insights into microglial roles in neurodegenerative diseases like Alzheimer’s disease, multiple sclerosis, and amyotrophic lateral sclerosis.

Here, we address this translational gap by comprehensively reconstituting the CNS microenvironment. We tested culture conditions incorporating *key niche-specific cytokines* (hTGF-β1, hIL-34, hCX3CL1) alongside *critical microenvironmental components*: extracellular matrix coatings (poly-L-ornithine/collagen IV), serum withdrawal, insulin-transferrin-selenium (ITS), cholesterol, urea and sodium selenite. We used quantitative real-time PCR to compare the expression of seven microglia-signature genes (*Tmem119*, *Cx3cr1*, *Hexb*, *P2ry12*, *Tgfbr1*, *Fcrl2* and *Olfml3*; see Table 1 for details) in freshly isolated and cultured microglia. These genes are expressed by CNS macrophages, but not by other myeloid cell populations. Additionally, we characterized the transcriptomes of freshly isolated and cultured microglia using DNA microarrays and RNA sequencing (RNA-seq).

**Table 1:** Homeostatic microglia signature genes examined in this study.

Gene	Full name	Function	Key references
*Tmem119*	Transmembrane protein 119	Type I transmembrane protein; used to distinguish microglia from infiltrating myeloid cells	Wickel 2024; Gosselin 2017
*P2ry12*	Purinergic receptor P2Y12	Gi-coupled ADP receptor; mediates ATP/ADP-guided process extension and chemotaxis supporting synapse surveillance	Wickel 2024; Kettenmann 2013; Gosselin 2017; Nimmerjahn 2005
*Cx3cr1*	C-X3-C motif chemokine receptor 1 (fractalkine receptor)	Chemokine receptor for Cc3cl1; regulates adhesion, migration, and neuron–immune communication	Cardona 2006; Paolicelli 2011; Wickel 2024
*Hexb*	Hexosaminidase subunit beta	Lysosomal enzyme subunit for degradation of glycoconjugates and gangliosides; supports lysosomal clearance of myelin/synaptic debris	Butovsky 2014
*Fcrl2*	Fc receptor-like S (scavenger receptor)	Ig superfamily-like orphan/scavenger-type receptor; likely contributes to scavenging/uptake	Butovsky 2014
*Olfml3*	Olfactomedin-like 3	Secreted extracellular matrix (ECM)-associated protein; may support ECM interactions and ramified morphology in microglia	Butovsky 2014; Haenseler 2017
*Tgfbr1*	Transforming growth factor beta receptor 1	Serine/threonine kinase receptor; mediates canonical Tgf-β signaling, essential for development and maintenance of microglial homeostatic identity (e.g., supports Tmem119/P2ry12 programs) and suppresses proinflammatory reprogramming	Butovsky 2014; Gosselin 2017; Haenseler 2017

Our systematic approach demonstrates that synergistic integration of these elements partially rescues homeostatic transcriptional programs lost during standard culture. This optimized protocol generates neonatal primary microglia with transcriptional and morphological features closer to ex vivo microglia controls, establishing a scalable platform for physiologically relevant disease modeling and mechanistic studies of murine microglia in vitro.

## Results

### Microglia isolation yields highly pure populations across workflows

**Fig. 1:**
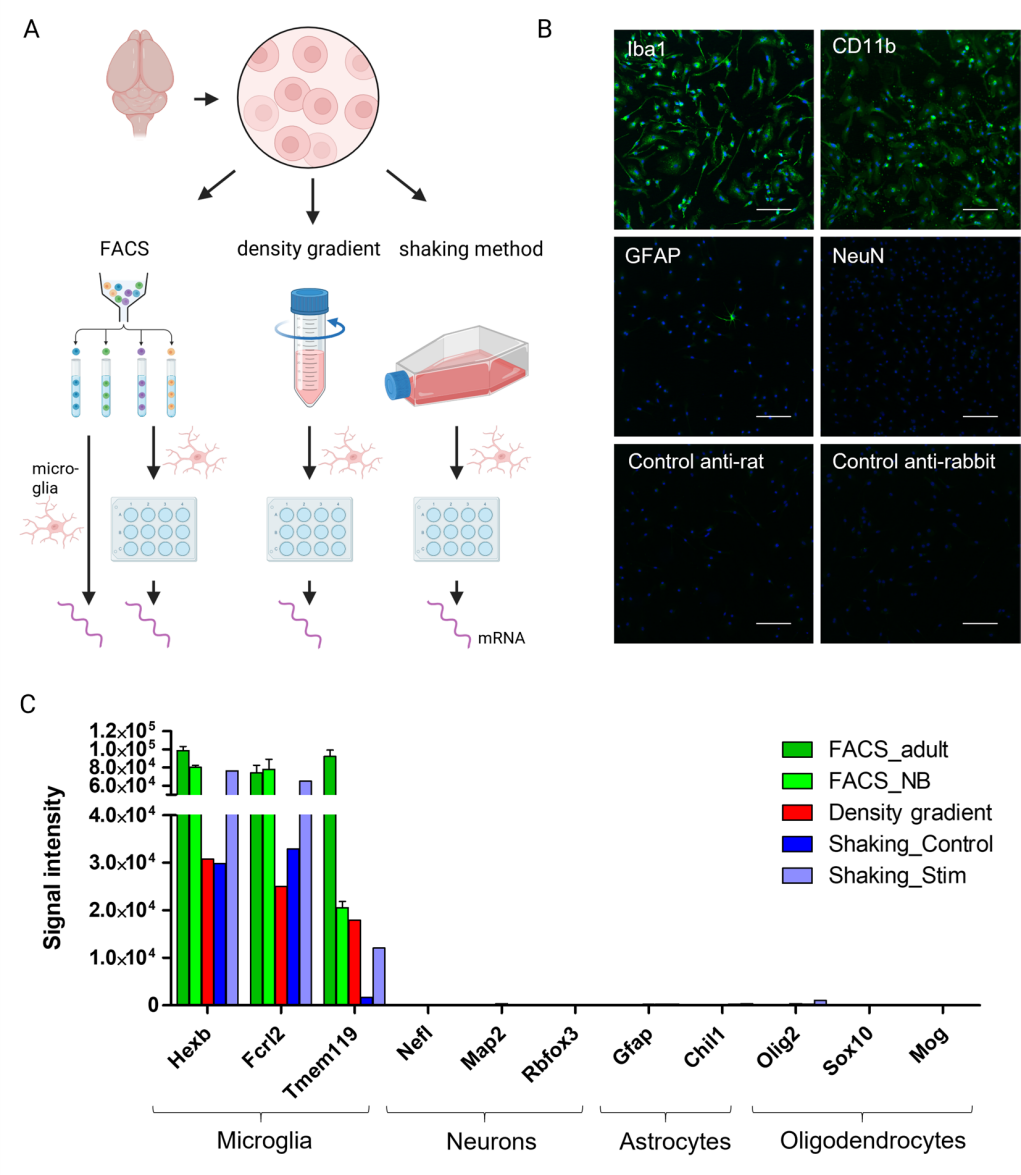
Microglia isolation. (**A**) Schematic representation of different methods used for microglia isolation. After obtaining cell suspensions from brains, microglia were isolated using either fluorescence-activated cell sorting (FACS), density gradient centrifugation, or the shaking method. mRNA was isolated from freshly isolated (FACS only) or cultured microglia. (**B**) Representative fluorescence images of isolated microglia using the shaking method. DAPI (blue) and marker proteins (green) specific for microglia (Iba1, CD11b), astrocytes (GFAP) and neurons (NeuN) were stained as indicated. Staining with secondary antibody only (control anti-rabbit, control anti-rat) served as a negative control. Scale bar: 100 μm. (**C**) Assessment of microglial purity based on microarray expression data. Bar plots display normalized signal intensities for five experimental groups across selected cell type–specific marker genes, including microglial (*Hexb*, *Fcrl2*, *Tmem119*), neuronal (*Nefl*, *Map2*, *Rbfox3*), astrocytic (*Gfap*, ), and oligodendrocytic (*Olig2*, *Sox10*, *Mog*) genes. Data are presented as mean ± standard deviation, illustrating the relative enrichment of microglial markers and the absence or low expression of markers from other central nervous system cell types.

To establish the fidelity of the starting microglial populations, we first compared three commonly used microglia isolation procedures (Fig. 1A) and assessed purity across workflows. After brain dissection and homogenization, microglia were separated using FACS sorting, density gradient centrifugation or the shaking method based on Barger and Basile^18^. Highly enriched populations of microglia were obtained using all three methods. For shaking-derived neonatal cultures, immunocytochemistry revealed strong immunoreactivity for the microglial markers ionized calcium-binding adaptor molecule 1 (Iba1; AIF1) and cluster of differentiation 11b (CD11b) and minimal detection of the astrocytic and neuronal markers glial fibrillary acidic protein (GFAP) and neuronal nuclei protein (NeuN; RBFOX3) at 24 h in vitro (Fig. 1B). In addition, analysis of our transcriptomic datasets showed very low expression of canonical neuronal (*Nefl*, *Map2*, *Rbfox3*), astrocytic (*Gfap*, *Chil1*) and oligodendrocytic (*Olig2*, *Sox10*, *Mog*) markers compared to microglial signature genes (*Hexb*, *Fcrls*, *Tmem119*) across the examined culture conditions (Fig. 1C), providing indirect evidence for high microglial enrichment. While these data demonstrate high microglial enrichment at the profiled time points, we cannot completely exclude that minor contamination by other brain-resident cell types may occur at later culture stages.

### Standard culture triggers rapid loss of homeostatic signature within 24 h

**Fig. 2:**
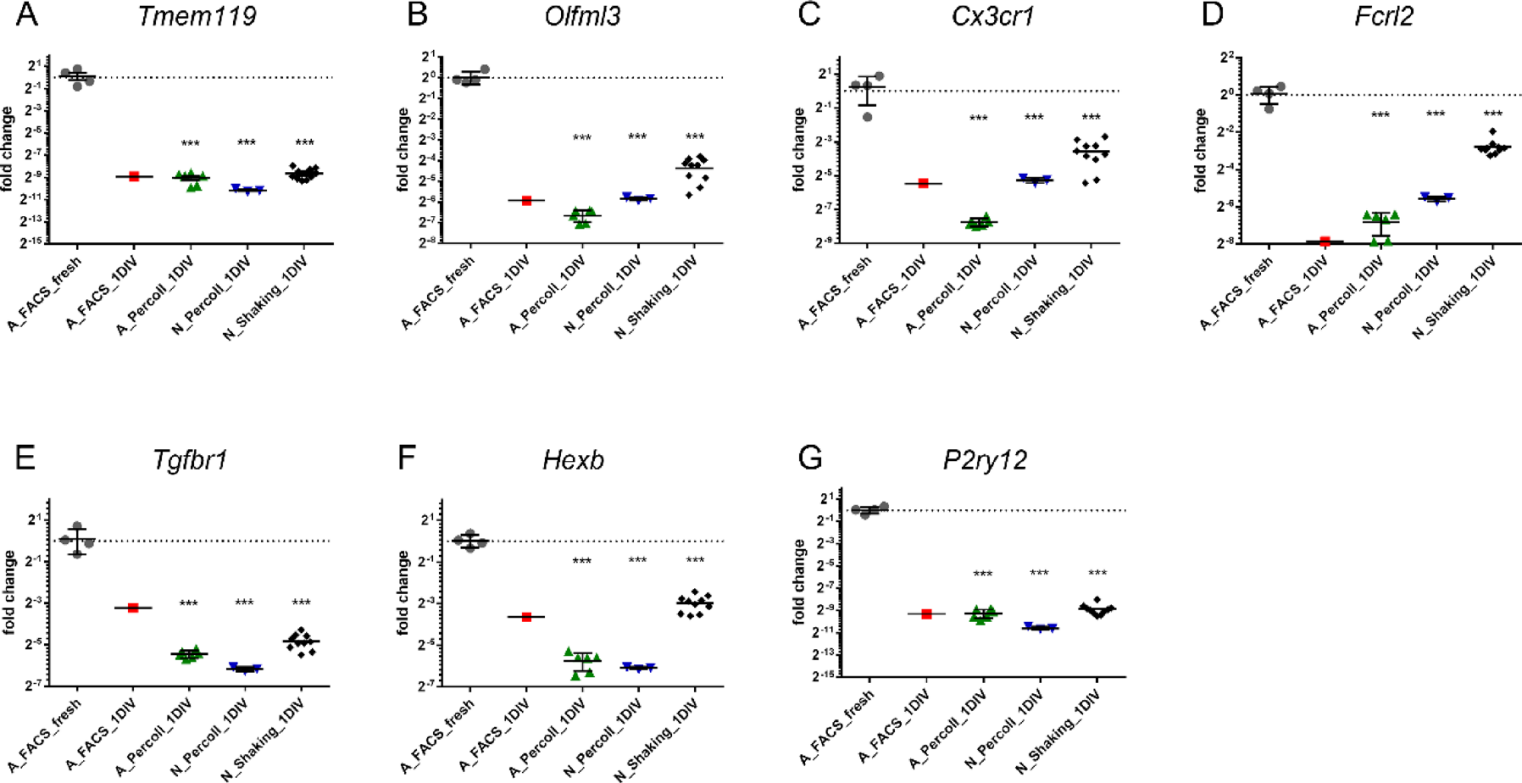
RT-qPCR analysis of seven microglial signature genes shows down regulation after in vitro cultivation. Microglia were isolated from adult (A, 22-week) or neonatal (N, postnatal day 1–3) C57BL/6N mice by: fluorescence-activated cell sorting (A_FACS_fresh, *n* = 4; A_FACS_1DIV, *n* = 1), density-gradient centrifugation (A_Percoll_1DIV, *n* = 7; N_Percoll_1DIV, *n* = 3), or shaking protocol (N_Shaking_1DIV, *n* = 10). RNA was isolated immediately after isolation (A_FACS_fresh) or after one day in vitro as monoculture (1DIV, all other groups). Data are presented as fold change (2 ^−ΔΔCt^) normalized to *Gapdh* and expressed relative to the mean of A_FACS_fresh; individual data points and mean ± SEM are shown. Statistics was performed on ΔCt values: one-way ANOVA with Dunnett’s multiple comparisons test vs. A_FACS_fresh. (**A**–**G**) individual genes as indicated. Significance: * *P* < 0.05; ** *P* < 0.01; *** *P* < 0.001.

Building on this validation, we next performed RT-qPCR to quantify seven canonical homeostatic transcripts (*Tmem119*,* Olfml3*,* Cx3cr1*,* Fcrl2*,* Tgfbr1*,* Hexb*,* P2ry12*) to test the hypothesis that in vitro culture precipitates a profound transcriptional reprogramming. We used freshly FACS-sorted microglia from 22-week-old female C57BL/6N mice as an ex vivo adult benchmark, against which cultured microglia from adult or neonatal mice were compared. The latter were derived via FACS, density gradient centrifugation and the shaking method and cultured in DMEM/F12 supplemented with 10% FBS and 50 µg/mL gentamicin. Irrespective of the isolation method and developmental stage, microglia cultured for 24 h as monoculture exhibited significantly reduced expression of all seven genes examined compared with freshly FACS-sorted adult microglia (Fig. 2A–G). This clearly shows that microglia rapidly lose their specific pattern of gene expression when deprived of their natural environment. Because our primary aim was to approximate adult microglial physiology in vitro, freshly sorted adult microglia were chosen as the reference baseline for these RT-qPCR analyses; neonatal ex vivo microglia were characterized in parallel at the transcriptome level (see Fig. 4A,B).

### Extended culture further erodes a subset of homeostatic transcripts

**Fig. 3:**
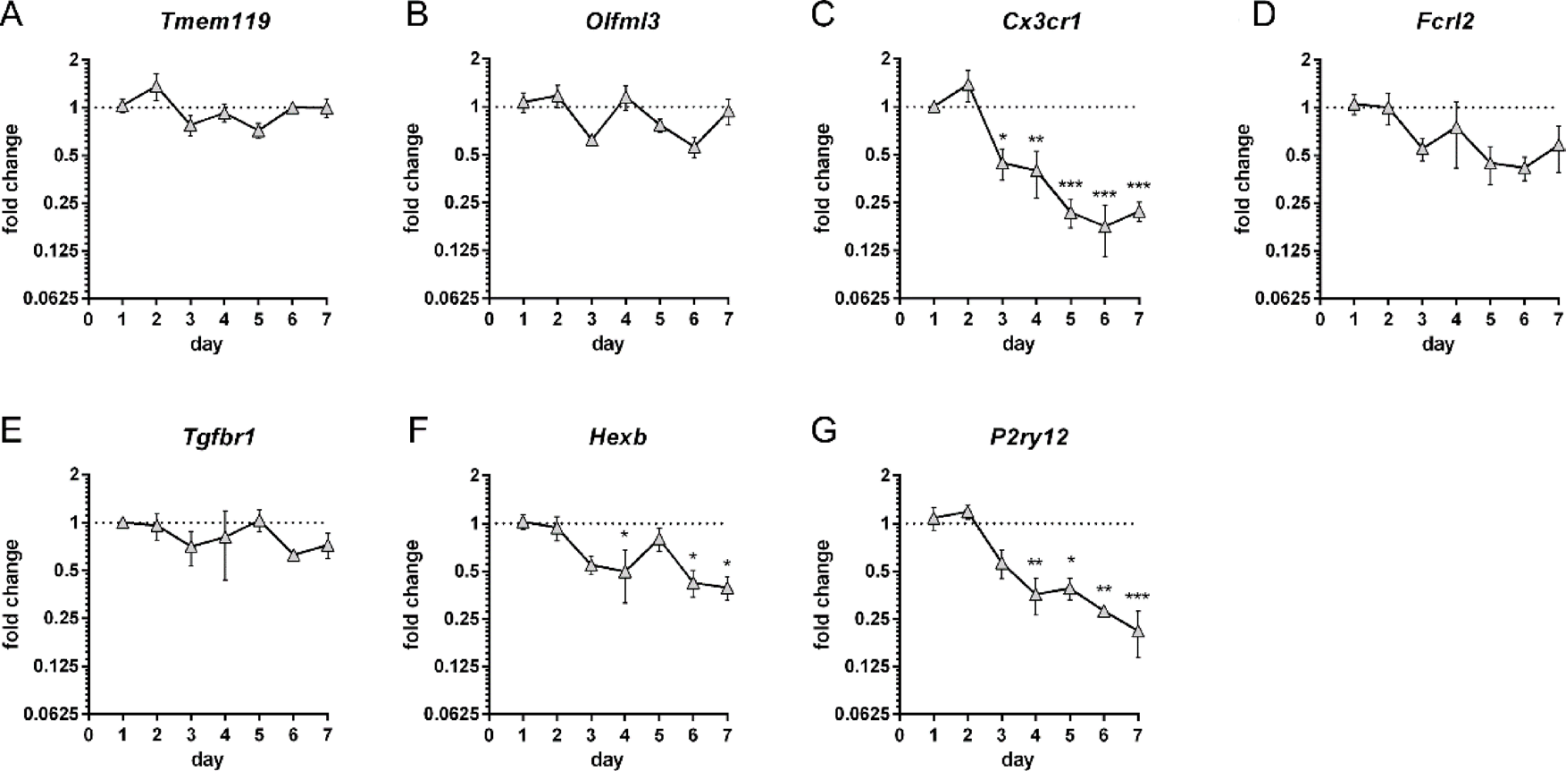
In vitro downregulation of specific microglia-enriched genes over time, analyzed by RT-qPCR. Microglia were isolated from neonatal C57BL/6N mice by shaking method and cultured for up to 7 days in 12-well plates in complete DMEM/F12. Data are presented as fold change (2^−ΔΔCt^) normalized to *Gapdh* and expressed relative to the mean of day 1, mean ± SEM. *n* = 7 for day 1, *n* = 3 for day 2–7. Statistical analysis was performed on ΔCt values: one-way ANOVA with Dunnett’s multiple comparisons test vs. day 1. Significance: * *P* < 0.05; ** *P* < 0.01; *** *P* < 0.001. (**A**–**G**), individual genes as indicated.

Building on this observation, we next examined whether extended culture duration elicits additional transcriptional changes. Over 7 days, three of the seven homeostatic genes (*Cx3cr1*,* Hexb*,* P2ry12*; Fig. 3C,F,G respectively) underwent a significant further decrease beyond the 24 h time point, whereas the remaining four transcripts (*Tmem119*,* Olfml3*,* Fcrl2*,* Tgfbr1*; Fig. 3A,B,D,E respectively) did not change significantly across the examined interval (Statistics: one-way ANOVA with Dunnett’s multiple comparisons test vs. day 1). Microglial signature gene expression can be restored when microglia are transplanted back into an intact brain, indicating that cultured microglia retain the capacity to recover their in vivo profile under appropriate conditions^8^. Consequently, in the next step, we aimed at counteracting the culture-induced loss of homeostatic gene expression in primary microglia by using stimulating factors normally present in the CNS.

### CNS niche cues (hTGF-β1, hIL-34, hCX3CL1) align cultured microglia with neonatal ex vivo profiles

Astrocyte-derived anti-inflammatory TGF-β plays an important role in maintaining microglial homeostasis^13,19^ and was used for culturing primary microglia by others^4,8^. In addition to hTGF-β1, we tested two other cytokines (hCX3CL1, hIL-34) that are primarily produced by neurons and are necessary for microglial survival and quiescence. To do so, we supplemented complete DMEM/F12 with different concentrations of hTGF-β1, hIL-34 and hCX3CL1 alone and in combination and assessed the expression fold change of seven homeostatic microglial genes. The increase in expression of these genes was mainly attributable to hTGF-β1 (Supplementary Fig. 1). However, all further experiments were conducted using a combination of all three cytokines at a concentration of 100 ng/mL each, in order to represent both astrocytic and neuronal influencing cues in vitro.

**Fig. 4:**
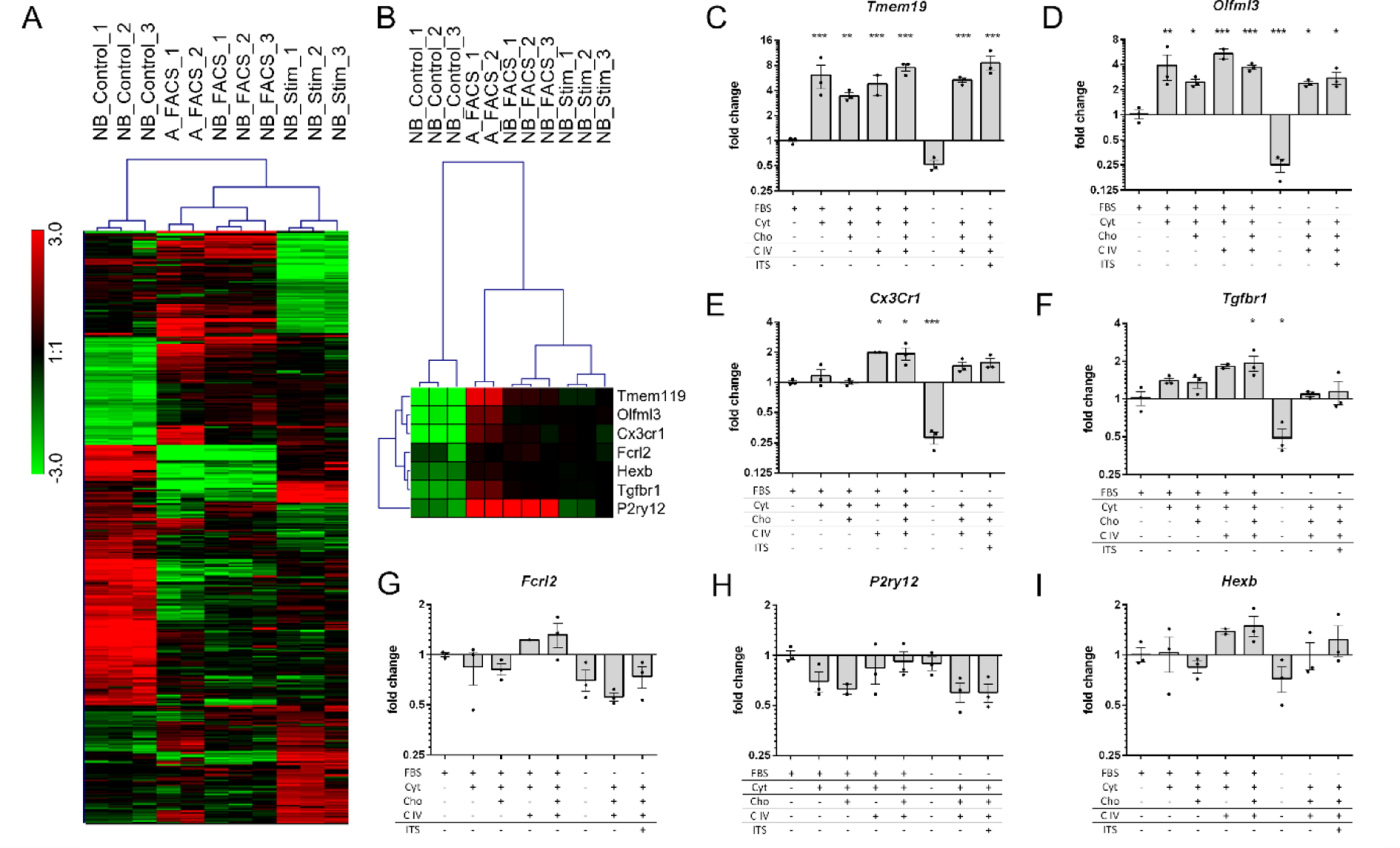
Influence of different cultivation conditions on microglial gene expression. (**A**) Heat-map of log2-transformed and median-centered differentially expressed genes in microglia from different sources based on microarray analysis: FACS-sorted, non-cultured microglia from adult C57BL/6N mice (A_FACS), FACS-sorted, non-cultured microglia from neonatal C57BL/6N mice (NB_FACS), microglia isolated from neonatal mice cultured for 1 day in DMEM/F12 + 10% FBS (NB_Control) or cultured in DMEM/F12 + 10% FBS + 100 ng/mL hTGF-β1 + 100 ng/mL hIL-34 + 100 ng/mL hCX3CL1 (NB_Stim). (**B**) Heat-map of log2-transformed and median-centered microglia marker genes in microglia from different sources (same as in A) based on microarray analysis. Probe sets from the same gene were mean-aggregated. Cluster analysis (Manhattan distance, complete linkage clustering) in A and B was performed with Genesis. (**C**–**I**) Quantitative RT-PCR of microglia isolated from neonatal C57BL/6N mice by shaking method and cultured for 24 h in 12-well plates in DMEM/F12 and varying medium supplements: 10% fetal bovine serum (FBS); 100 ng/mL hTGF-β1, 100 ng/mL hIL-34, 100 ng/mL hCX3CL1 (Cyt); 1.5 µg/mL cholesterol (Cho); 0.1x insulin-transferrin-selenium solution (ITS). Well plates were coated with 2 µg/mL collagen-IV (C IV) before seeding cells as indicated. Data are presented as fold change (2^−ΔΔCt^) normalized to Gapdh and expressed relative to the mean of the control (DMEM/F12 + FBS), mean ± SEM. *n* = 1–3. Statistical analysis was performed on ΔCt values: One-way ANOVA with Tukey’s multiple comparisons test, only *P*-values for comparisons vs. control are shown. (**C**–**I**) individual genes as indicated. Significance: * *P* < 0.05; ** *P* < 0.01; *** *P* < 0.001.

We performed microarray analyses to further characterize differences in gene expression between freshly isolated adult and neonatal microglia, which served as ex vivo reference samples for two developmental stages, and cultured microglia. As shown in Fig. 4A, the gene expression profile of untreated cultured microglia clearly discriminated these cells from all other samples. In contrast, microglia cultures stimulated with the cytokine cocktail described above showed a gene expression pattern that placed these cells closer to freshly isolated microglia, suggesting that the stimulation had shifted the gene expression profile toward that of FACS-sorted microglia. Similar results were observed in the analysis of the seven microglia-specific genes, with the distinction that the treated microglia clearly clustered together with freshly isolated microglia from neonates (Fig. 4B). This finding is consistent with expectations, as the cultured microglia were originally derived from neonatal mice.

### Extracellular matrix and lipid cues (Collagen IV, Cholesterol) fine-tune homeostatic genes

We further reconstituted the CNS niche beyond canonical cytokines. Therefore, we assessed the influence of culture vessel coatings (poly-L-ornithine, collagen IV), the use of serum-free medium, and defined media supplements – cholesterol, urea, sodium selenite, and insulin-transferrin-selenium (ITS) solution – on microglial transcription using RT-qPCR. Figure 4C–I depicts the impact of selected culture conditions on the expression of seven homeostatic microglial genes. In line with the microarray analyses, supplementation with three cytokines (hTGF-β1, hIL-34, hCX3CL1) significantly increased *Tmem119* and *Olfml3* expression by 6.1-fold and 3.9-fold, respectively, relative to cytokine-free medium (Fig. 4C,D; one-way ANOVA with Tukey’s multiple comparisons test). For , the increase in fold change reached significance through the use of 2 µg/mL collagen IV as a vessel coating in addition to cytokines (Fig. 4E). For *Tgfbr1*, significant increase in fold change was achieved by additionally adding 1.5 µg/mL cholesterol to the culture medium (Fig. 4F). The examples show that certain microglial genes can be increased by adding cytokines to the culture medium, and that this effect can be enhanced by collagen IV and cholesterol for individual genes. Neither poly-L-ornithine coating nor media supplementation with urea or sodium selenite could further increase the expression of the genes examined in addition to the cytokines (Supplementary Fig. 2).

Serum withdrawal (FBS-free medium) reduced expression across all genes, reaching significance for *Olfml3*,* Cx3cr1*, and *Tgfbr1* compared with DMEM/F12 containing 10% FBS (Fig. 4D,E,F). This deficit was largely rescued by collagen IV coating together with cytokines, cholesterol and ITS-supplementation, restoring expression to levels comparable to FBS-supplemented standard medium for most genes (except *Fcrl2* and *P2ry12*) and even surpassing baseline significantly for *Tmem119* and *Olfml3*. ITS was used at this point to partially replace serum by providing defined metabolic support.

### Serum-free, collagen IV–based conditions with cholesterol preserve ramified morphology and enhance transcriptional recovery

**Fig. 5:**
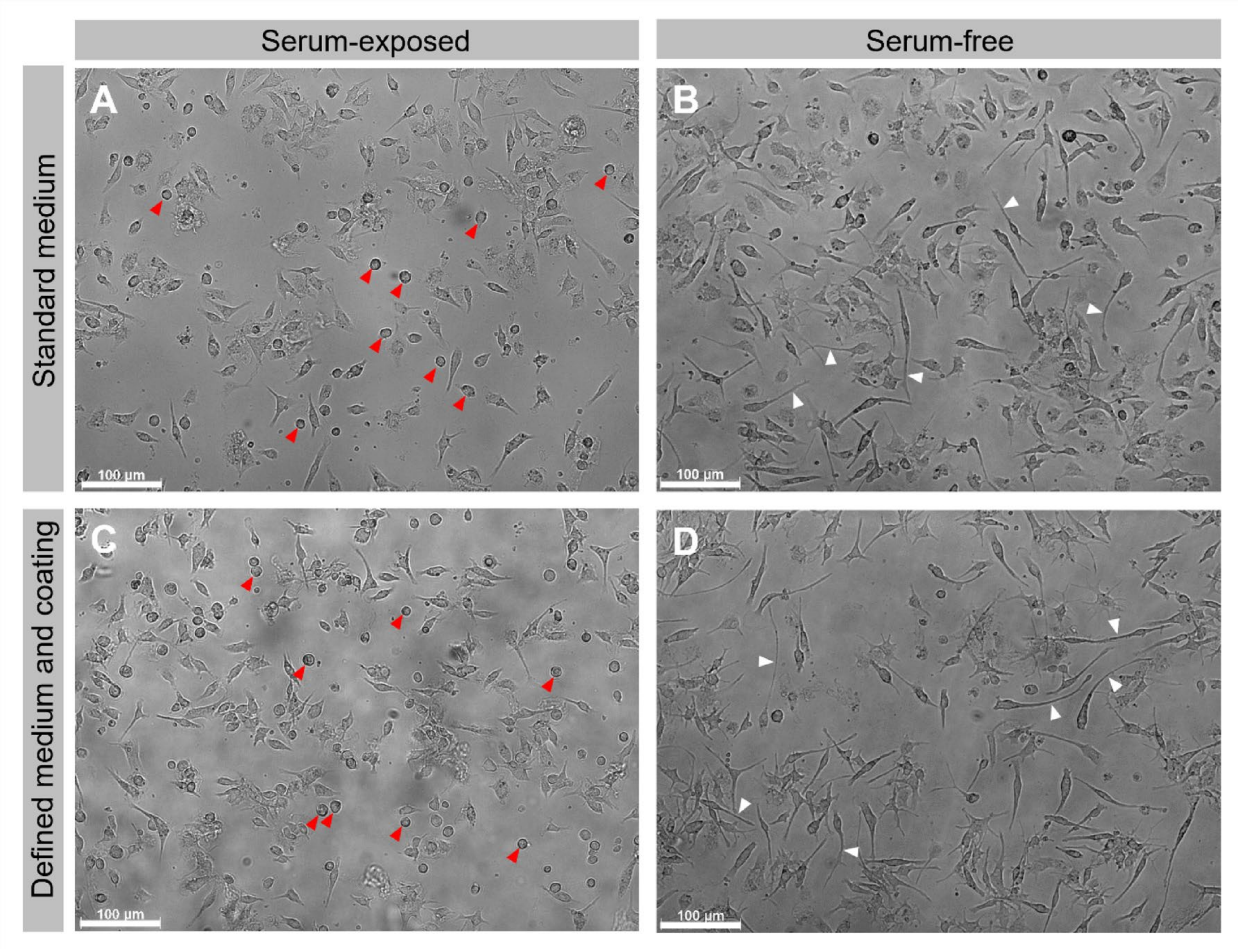
Morphological changes in microglia upon culture in serum-free medium. Primary C57BL/6N microglia were cultured in serum-containing (**A**, **C**) or serum-free (**B**, **D**) glial medium (DMEM/F12 + 50 µg/mL gentamicin). Tissue culture plates in (**C**) and (**D**) were coated with collagen IV (2 µg/mL) and medium was supplemented with hIL-34, hTGF-β1, and hCX3CL1 (100 ng/mL each) and cholesterol (1.5 µg/mL). Red arrowheads point to round microglia without processes, which are typical of an activated state. White arrowheads point to long, thin microglia processes, which are characteristic of a resting phenotype. Representative images for each condition are shown (×20 magnification). Scale bar: 100 μm.

Serum profoundly alters the microglial phenotype in vitro, driving amoeboid morphology, proliferation, and heightened phagocytosis (Bohlen et al. 2017). Although serum withdrawal reduced expression of all genes examined, it was associated with a more ramified morphology characterized by long, thin processes typical of homeostatic (“resting”) microglia (Fig. 5B,D). In contrast, microglia cultured with serum exhibited predominantly amoeboid or rounded cell bodies with few thick processes, consistent with an “activated” state (Fig. 5A,C). Importantly, supplementation of serum-free cultures with defined factors (cytokines and cholesterol) on collagen IV largely restored microglial signature gene expression while preserving ramified morphology. Together, these findings support serum-free culture, combined with defined supplements, as a preferred condition to approximate a resting-like microglial phenotype in vitro based on morphology and homeostatic gene expression (see Table 2 for medium recipe).

### Optimized culture conditions improve microglial homeostatic gene expression signature

**Fig. 6:**
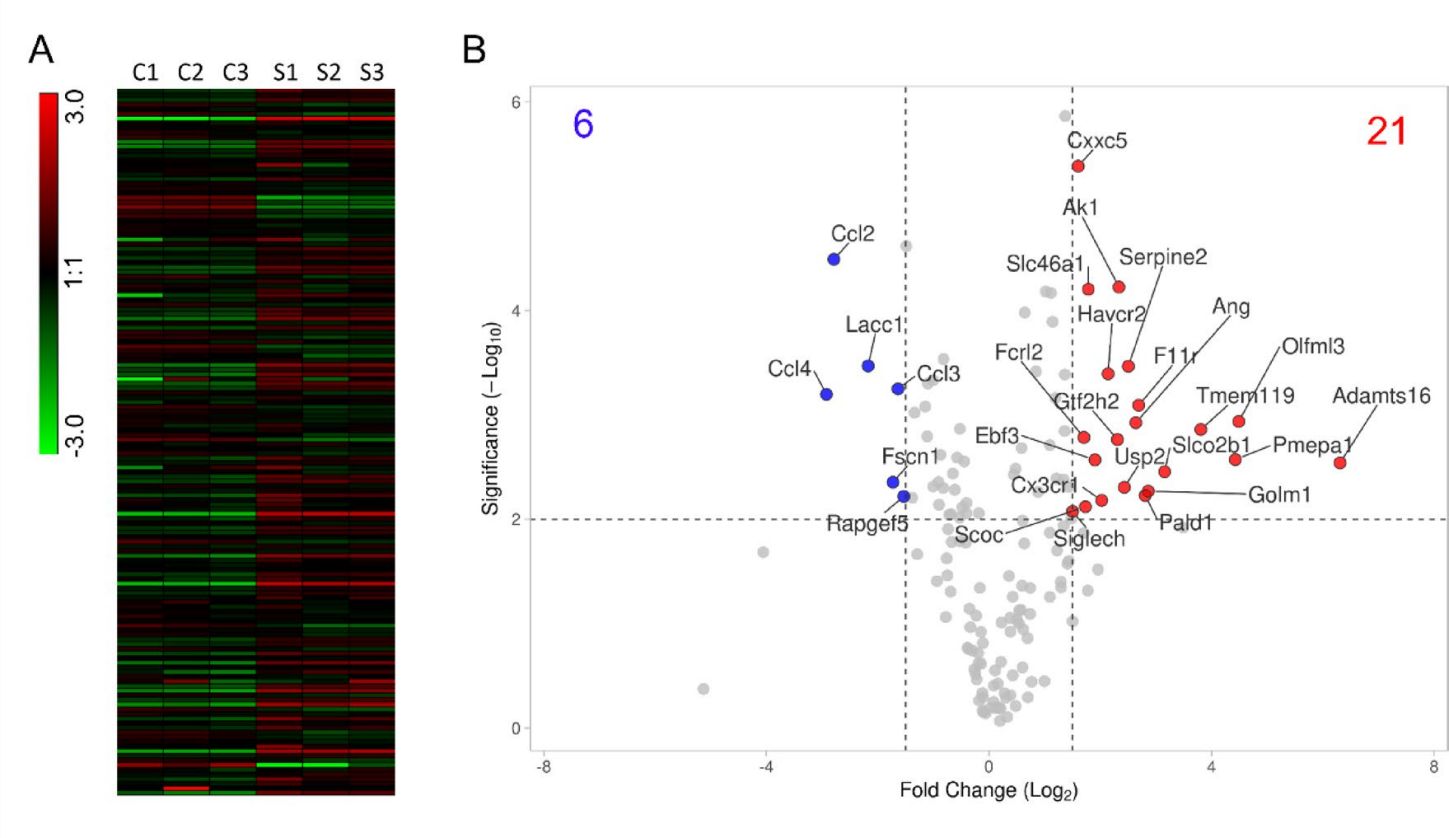
RNA-seq analysis reveals that expression of most microglia-specific homeostatic genes are increased by optimized medium and coating. (**A**) Heat map of 152 microglial specific genes (according to Butovsky et al., 2013) differentially expressed between microglia cultured in serum-free DMEM/F12 (control, C1-3) and microglia cultured in serum-free DMEM/F12 supplemented with hIL-34, hTGF-β1, and hCX3CL1 (100 ng/mL each), cholesterol (1.5 µg/mL) and 0.1× insulin-transferrin-selenium solution on collagen IV-coated culture wells (S1-S3). Three biological replicates are shown per condition. (**B**) Volcano plot showing differential expression of 152 microglial specific genes in microglia cultured in serum-free DMEM/F12 compared with microglia cultured under defined conditions as described in (A) for S1-3. Values in the upper corners indicate the number of upregulated (red) and downregulated (blue) genes.

Butovsky et al. defined a set of 152 microglia-specific homeostatic genes^4^. To assess how medium supplements and vessel coatings influence their expression in culture, we performed RNA sequencing of microglia under combined, optimized (as reported in Table 2) versus control (serum-free DMEM/F12) conditions (Fig. 6). Under optimized conditions, most of the 152 genes were upregulated compared with controls (Fig. 6A), resulting in an expression profile that shifted toward the ex vivo pattern observed in freshly FACS-sorted microglia. The volcano plot identified 21 genes with a significant (*p* < 0.01) |log2 fold change|≥1.5, including *Tmem119*, *Olfml3*, *Cx3cr1* and *Fcrl2* discussed above. Other upregulated genes included for example *Havcr2*,* Pmepa1* and *Serpine2*. *Havcr2*, also known as *Tim3*, encodes a Th1-specific cell surface protein and is induced by TGF-β signaling. HAVCR2 was shown to suppress immune cells and inhibit phagocytic behavior of microglia^20^. Another TGF-β-inducible gene encodes prostate transmembrane protein, androgen induced 1 (PMEPA1), that acts as a negative feedback regulator of TGF-β signaling by promoting receptor degradation and limiting SMAD activation^21^. SERPINE2, a serine protease inhibitor, regulates extracellular proteolysis and inflammatory signaling. Its deletion alters antimicrobial gene expression in microglia, suggesting a role in immune homeostasis^22^.

Conversely, only six genes were downregulated. Each is linked to inflammatory activation: pro-inflammatory CC chemokine ligands (CCL) are associated with sustained microglial activation^23^; Laccase domain-containing 1 (LAAC1) mediates endoplasmic reticulum stress and exacerbates neuroinflammation^24^; Fascin-1 (FSCN1) is upregulated upon myelin-debris stimulation, coinciding with enhanced microglial migration^25^; and Rap guanine nucleotide exchange factor (RAPGEF) enhances microglial phagocytosis via RAP1 activation^26^. Their downregulation under optimized culture conditions indicates that supplementation represses inflammatory genes and promotes a less activated, more homeostatic transcriptional profile. The observed gene expression changes reflect the combined optimized condition; we did not isolate the independent contribution of single cytokines or supplements to these RNA-seq outcomes.

## Discussion

In this study we showed that standard in vitro culture of primary mouse microglia precipitates a rapid and profound loss of homeostatic features within 24 h of plastic adherence, as evidenced by downregulation of signature transcripts (*Tmem119*,* P2ry12*,* Cx3cr1*,* Hexb*,* Fcrl2*,* Olfml3*,* Tgfbr1*). Critically, this “culture shock” can be partially reversed by reconstituting key CNS niche signals – hTGF-β1, hIL-34, and hCX3CL1 – together with collagen IV coating, cholesterol and insulin-transferrin-selenium in serum-free conditions, yielding both transcriptional recovery (particularly *Tmem119*,* Olfml3*,* Cx3cr1*,* Tgfbr1*) and ramified morphology consistent with a more resting-like microglial state. These results address the translational gap outlined in the introduction by demonstrating that microenvironmental inputs essential for microglial homeostatic features in vivo can be leveraged to partially preserve these features in primary microglia ex vivo^6,7,27^. We used neonatal primary microglia and therefore confine our conclusions to neonatal-derived cultures. Although developmental stage shapes baseline transcription, the direction of cytokine and ECM responses aligns with reports in adult microglia^4,8^.

An important control in our study was the comparison across isolation methods – FACS, Percoll, and shaking – which all converged on the same rapid loss of homeostatic transcripts after 24 h, whereas freshly FACS-sorted adult microglia maintained high expression of signature genes at baseline. This points to culture context as the dominant driver of transcriptional drift, rather than the isolation workflow per se, consistent with environment-dependent microglial identity^7^. Over a 7-day time course, we observed further progressive reductions for a subset of genes (*Cx3cr1*,* Hexb*,* P2ry12*), suggesting a continuing adaptation to the artificial culture milieu and highlighting the need for sustained niche support to prevent phenotypic erosion^7,8^.

Because the ultimate goal was to approximate adult microglial physiology in vitro, particularly in the context of neuroinflammation and neurodegeneration, we used freshly isolated adult microglia as the reference baseline for our RT-qPCR analyses. Neonatal microglia are technically more accessible and yield higher cell numbers, but they differ from adult microglia in their baseline transcriptional, morphological, and functional properties. We therefore included neonatal ex vivo microglia as a separate reference at the transcriptome level (Fig. 4A,B) and interpret our data as assessing how far neonatal-derived cultures can be driven toward adult-like homeostatic transcriptional signatures, rather than implying equivalence between neonatal and adult microglia.

Our observations are congruent with prior work showing that microglial phenotype is tightly governed by CNS-derived cues and is rapidly reprogrammed upon removal from their tissue context^4,8^. Immediate ex vivo profiling revealed that microglia possess a distinct homeostatic transcriptional network in vivo which collapses in conventional culture, with downregulation of *Tmem119* and *P2ry12* and induction of activation and proliferative programs^7,27^. Serum-containing media are a major driver of this drift, promoting amoeboid morphology, proliferation, heightened phagocytosis, and broad activation signatures^8,28^. In line with these reports, we found that serum exposure favored amoeboid microglia, whereas microglia cultured under serum-free conditions yielded thin, highly branched processes typical for surveillant microglia. However, serum withdrawal alone reduced signature gene expression, underscoring that phenotype stabilization requires both removal of activating stimuli and active provision of pro-homeostatic signals. We used insulin-transferrin-selenium (ITS) to partially replace serum by providing defined metabolic support and maintaining baseline viability. Selenium, supplied as part of ITS, is essential for antioxidant defense in microglia, especially through its role in glutathione peroxidase 4 (GPX4), protecting cells from oxidative stress and ferroptosis^29,30^. Insulin regulates microglial glucose uptake and homeostatic metabolism primarily via IRS-1/PI3K/AKT signaling pathways, which are crucial for maintaining microglial quiescence and function^31,32^. Transferrin ensures sufficient iron availability for cell proliferation and enzymatic reactions; limiting free iron is also neuroprotective^33,34^. We did not directly quantify proliferation or phagocytic activity in our cultures, so our conclusions are limited to transcriptional and morphological endpoints.

Building on prior work that used CNS niche cytokines to stabilize primary microglia^4,8^, our data indicate that hTGF-β1 is the predominant driver of transcriptional rescue, while hIL-34 and hCX3CL1 were included to represent neuronal survival and restraint signals. The three-factor mix improved morphology and shifted global profiles toward neonatal ex vivo microglia, without implying additive or dominant effects of hCX3CL1 or hIL-34. Specifically, TGF-β signaling is indispensable for microglial development and maintenance of the homeostatic signature (including *Tmem119* and *P2ry12*) in vivo and can restore aspects of microglial identity ex vivo^19^. The canonical Smad-dependent TGF-β pathway acts as a central regulator of microglial maturation, ensuring their differentiation from progenitors and sustaining a quiescent, surveillant phenotype under physiological conditions^19^. Genetic ablation of TGF-β signaling in microglia leads to a loss of homeostatic markers and induces a transcriptomic shift toward a disease-associated or primed state, highlighting its role in preventing maladaptive activation^19^. IL-34, a high-affinity ligand for CSF1R, is a key survival factor for microglia, and sustained CSF1R signaling is necessary for microglial viability and appropriate transcriptional programs^35,36^. The neuronal chemokine CX3CL1 (fractalkine) restrains microglial activation via CX3CR1 and modulates microglia–synapse interactions, thereby supporting surveillance and limiting inflammatory responses^37,38^. Furthermore, protocols for human iPSC-derived microglia typically combine IL-34 and TGF-β to recapitulate core aspects of microglial identity, supporting the translational relevance of our cytokine cocktail approach^10,39,40^. Together, these literature data provide a biological rationale for the restoration of signature transcripts we observed when hTGF-β1, hIL-34, and hCX3CL1 were supplied.

Beyond these soluble cues, additional contributions from extracellular matrix shaped the magnitude of rescue: we observed that coating of cell culture wells with 2 µg/mL collagen IV augmented the effects of the cytokine cocktail on selected microglial genes and preserved the ramified morphology. Collagen IV is a principal component of vascular basement membranes, that microglia engage via integrin-dependent focal adhesions, thereby modulating their adhesion, mechanosensation, and directed motility at the blood-brain barrier^41^. Accordingly, presenting collagen IV in vitro may help sustain the adhesion, survival, and homeostatic phenotype of primary microglia by mimicking the basement membrane environment of the neurovascular unit and providing integrin-mediated signaling cues that support ramified morphology and anti-inflammatory functions^41^. Besides that, cholesterol availability is a key determinant of microglial identity: microglia sense neuronal cholesterol via Trem2, a receptor essential for microglial metabolic fitness, which regulates ApoE-dependent lipid processing^42^. However, local excessive amounts of cholesterol in the CNS, for example during the breakdown of myelin, can lead to cholesterol transport capacity being overwhelmed. This can induce stress responses in microglia and cause a disease-associated transcriptional state^43^. In this study, 1.5 µg/mL cholesterol did not increase the majority of homeostatic genes examined and affected *Tgfbr1* only in the presence of cytokines and collagen IV. We did not measure survival directly, and inclusion was based on prior reports of improved viability^8^.

Experimental approaches exist in which primary microglia are cultivated alongside astrocytes, neurons, or both^44–46^. Factors secreted by these cells into the culture medium support the ramification of the microglia and induce the expression of genes that are typically lost in vitro. The disadvantage of these approaches is that they are costly and time-consuming to set up, as up to three different cell types must be provided. By contrast, our protocol enables the easy and rapid addition of selected CNS niche cues, offering a simplified solution that can produce similar beneficial effects on cultured microglia morphology and homeostatic gene expression, although we did not directly compare our conditions to such co-culture systems.

Several conceptually related studies have previously combined CSF1R ligands, TGF-β and cholesterol in serum-free conditions to support rodent^8^ or human^47,48^ microglia. Our work builds on these protocols but extends them in three main ways. First, we systematically quantified a defined set of homeostatic genes by RT-qPCR across multiple isolation methods and culture conditions, demonstrating that the same rapid culture-induced loss and partial rescue are observed regardless of the initial isolation workflow. Second, we validated the impact of the optimized condition transcriptome-wide using microarray and RNA-seq, showing that most genes from the Butovsky homeostatic gene set are upregulated and that inflammatory and stress-associated programs are attenuated relative to serum-free control medium. Third, we provide a practical, collagen IV–based, chemically defined, serum-free recipe that can be implemented without co-culture and is specifically optimized for neonatal primary microglia. In particular, whereas Bohlen et al. primarily characterized microglial survival, morphology and phagocytic capacity under defined serum-free conditions, our data provide the complementary genome-wide transcriptional landscape of microglia exposed to conceptually similar CNS niche cues, thereby linking functional and molecular readouts and offering an experimentally accessible workflow for stabilizing homeostatic-like signatures in primary culture.

Several limitations should be considered when interpreting our findings. First, microglial enrichment was assessed by immunocytochemistry for shaking-derived cultures at early time points and by transcriptomic analysis of lineage marker expression in selected conditions, which indicated very low expression of neuronal, astrocytic and oligodendrocytic markers relative to microglial signature genes. However, we did not systematically quantify contamination across all isolation methods, conditions and time points, so minor contributions from other brain-resident cell types cannot be completely excluded. Second, our ex vivo reference transcriptomes were obtained after enzymatic digestion in the presence of FBS and without transcriptional inhibitors such as actinomycin D, and therefore likely already reflect some isolation-induced changes; they should be interpreted as approximations to the in vivo state rather than undisturbed in vivo profiles. Third, our primary readouts were mRNA expression and morphology. We did not directly assess functional endpoints such as phagocytic capacity, cytokine secretion or proliferation, and therefore do not claim that these aspects of microglial function are fully preserved under our optimized conditions. Future studies should complement our transcriptomic and morphological analyses with functional assays (e.g., phagocytosis assays, cytokine secretion measurements, and Ca²⁺-signaling or calcium-imaging readouts) to determine to what extent the optimized serum-free, collagen IV–based conditions also preserve microglial functions relevant to neuroinflammation and CNS homeostasis. Fourth, RT-qPCR normalization relied on single reference genes (*Gapdh* or *Ywhaz*), whose expression can be modulated by activation state and culture condition; although the major expression trends observed by RT-qPCR were consistent with those from microarray and RNA-seq, the microarray and RNA-seq datasets were normalized using global approaches that do not rely on individual housekeeping genes and therefore provide an independent reference for the direction of expression changes. The use of single housekeeping genes in our RT‑qPCR assays nevertheless represents a methodological limitation that should be addressed in future work by validating multiple reference genes.

In summary, we provide evidence that the rapid loss of microglial homeostatic features after plastic adherence is not irreversible: a defined set of CNS niche cues – hTGF-β1, hIL-34, and hCX3CL1 – combined with ECM and lipid support, can partially restore homeostatic-like transcriptional signatures and ramified morphology in cultured neonatal primary microglia. This optimized, scalable workflow is expected to improve physiological relevance in vitro compared with conventional serum-containing culture, mitigate culture-induced transcriptional artifacts, and enable more robust interrogation of microglial mechanisms implicated in neuroinflammation and synapse regulation across disease models.

## Methods

### Animals

Breeding pairs of mouse strain C57Bl/6N were purchased from Janvier Labs (Le Genest Saint Isle, France). Neonates and adult mice used in this study were bred at Fraunhofer IZI, Halle (Saale), Germany. Mice were maintained in individually ventilated cages (IVC, type II long, Tecniplast) with maximum of 5 mice per cage at the facility of Fraunhofer IZI in Halle (Saale), Germany. The housing conditions (room temperature: 22 °C ± 2 °C, humidity: 50% ± 20%) were controlled by a central ventilation system. The light/dark cycle (12 h day / 12 h night) was regulated. Primary microglia were isolated from postnatal day (P1–P3) neonates of both sexes (average weight 1–2 g) and from adult female mice aged 12–24 weeks (average weight 25–30 g). Neonates were euthanized by decapitation. Adult mice were sacrificed via inhalation of 100% CO_2_ in a designated home cage followed by transcardial perfusion with ice-cold Hanks’ Balanced Salt Solution (HBSS).

### Ethics declaration

A formal approval by an animal ethics committee was not required for this study, and an ethics approval waiver applies under the relevant German regulations. This study did not involve any experiments on live vertebrate animals. All mice were killed solely for the purpose of organ (brain) collection by trained personnel in accordance with Section  4(3) of the German Animal Welfare Act. Under the German Animal Welfare Act implementing Directive 2010/63/EU, the killing of animals exclusively for the use of their organs or tissues, without any prior intervention for experimental purposes, is not classified as an animal experiment and is therefore exempt from project authorization and formal ethics committee approval (Sections  7–8, German Animal Welfare Act). The numbers of mice killed for organ harvesting were reported annually to the competent authority (Landesverwaltungsamt Halle, Halle (Saale), Germany) as required by German law. We confirm that all procedures and animal handling were carried out in full compliance with the German Animal Welfare Act, Directive 2010/63/EU, and recommendations from the Federation of European Laboratory Animal Science Associations (FELASA).

### Brain dissection and dissociation

Brains were harvested under sterile conditions. Tissue digestion was performed using 0.25% trypsin in DMEM/F12 medium (Gibco) supplemented with 10% fetal bovine serum (FBS, Gibco) and 50 µg/mL gentamicin (called “complete DMEM/F12” in the following) for 15 min at 37 °C. Subsequently, DNase I (Roche, 0.4 mg/mL in DMEM/F12) was added and incubated for 5 min at room temperature. Adult brains were homogenized by gently pressing the tissue through a 100 μm cell strainer (Greiner Bio-One) using a sterile syringe plunger, with repeated rinsing using DNase-containing complete DMEM/F12 (approximately 20 mL per brain). Neonatal brains were dissociated using a 10 mL serological pipette followed by a 1 mL pipette, and the resulting suspension was filtered through a 100 μm cell strainer. Cell suspensions were centrifuged at 300 ×*g* for 10 min. Supernatants were discarded, and pellets were resuspended in 10 mL of complete DMEM/F12. The suspension was further filtered through a 40 μm cell strainer and centrifuged again under the same conditions. The resulting cell pellet was subsequently processed for microglia isolation using either fluorescence activated cell sorting (FACS), Percoll gradient centrifugation or mechanical shaking. The use of 10% FBS during enzymatic digestion and washing steps was chosen in line with commonly used protocols to support cell viability during tissue dissociation; we acknowledge that this transient exposure may already influence microglial transcriptional profiles, and this limitation is considered in the interpretation of ex vivo control conditions.

### Fluorescence-activated cell sorting

FACS was performed according to the protocol of Pösel et al.^49^. Briefly, cell suspensions obtained from adult and neonatal C57BL/6N mice were subjected to a 25% Percoll centrifugation to remove myelin. Subsequently, cells were blocked for 10 min at 4 °C with CD16/CD32 Fc-receptor blocking agent (final concentration 2.5 µg/mL, Invitrogen) and stained simultaneously with a PE-conjugated CX3CR1 antibody (1:10, R&D Systems, FAB5825P-025) and an APC-conjugated CD45.2 antibody (1:40, Thermo Fisher Scientific, 17-0454-82) for 25 min at 4 °C in the dark. After washing, cells were resuspended in FACS buffer (3% FBS in phosphate-buffered saline (PBS)). Based on intermediate CD45 signal and high CX3CR1 signal highly pure microglia were sorted into FACS buffer using a FACSAria II (BD Biosciences). Approximately 100.000 microglia cells were sorted from one adult brain, while 40.000 microglia cells were obtained from one neonatal brain. After sorting, cells were centrifuged for 5 min at room temperature at 400 ×*g*, the pellet was resuspended directly in lysis buffer for RNA isolation or in complete DMEM/F12 for subsequent culturing.

### Microglia isolation using percoll gradient centrifugation

Cell pellets obtained from adult and neonatal mouse brains were resuspended in 5 mL PBS and centrifuged at 500 ×*g* for 10 min. For density gradient centrifugation, colloidal silica (Percoll, GE Healthcare) was employed. A stock of isotonic Percoll solution (SIP) was prepared by mixing 9 parts Percoll with 1 part 1.5 M NaCl. SIP was subsequently diluted with PBS to generate 70% and 30% SIP working solutions. The cell pellet was resuspended in 10 mL of 70% SIP and carefully overlaid with 10 mL of 30% SIP, followed by an additional 5 mL of PBS layered on top. The gradient was centrifuged at 300 ×*g* for 40 min at room temperature, with low acceleration and without brakes. The myelin layer, located at the interface of the 30% SIP and PBS, was aspirated. The cell layer at the interface between the 70 and 30% SIP phases was carefully collected and transferred to a new centrifuge tube. Cells were washed by diluting the suspension to 50 mL with PBS and centrifuged at 300 ×*g* for 10 min. The resulting pellet was resuspended in 500 µL complete DMEM/F12.

### L-929 conditioned medium

To obtain macrophage colony-stimulating factor (M-CSF)-rich conditioned medium for microglia proliferation, L-929 cells (DSMZ, Braunschweig, Germany^50^ were cultured adherently in RPMI 1640 medium (Gibco) supplemented with 10% FBS in 75 cm² tissue culture flasks. Conditioned medium was collected every 2–3 days and replaced with fresh medium. Harvested medium was sterile-filtered using 0.2 μm syringe filters and stored at −20 °C until use.

### Microglia Isolation using Shaking Protocol

Microglia isolation was performed using the shaking protocol as described by Barger and Basile^18^ with minor adaptations. The cell suspension of 2–3 neonatal brains was seeded in complete DMEM/F12 in a 75 cm^2^ cell culture flask coated with 0.1% poly-L-ornithine (PLO, Gibco). Flasks were washed with complete DMEM/F12 after overnight incubation and stimulated with 20% L-929 conditioned medium in complete DMEM/F12 on day 8, 13, 21 and 29 after isolation. Microglia were collected by shaking the flasks for 5.5 h at 70 rpm and subsequently aspirating the cell suspension on days 18, 26 and 34 after isolation. After collection of microglia, the glia cell culture was replenished with complete DMEM/F12 (+ 20% L-929 conditioned medium) and incubated for the next shaking isolation. Cultures were discarded after the 3rd harvest on day 34.

### Primary microglia stimulation

Highly pure microglia obtained by FACS, density gradient centrifugation or the shaking protocol, were seeded at a density of 0.5 or 1 × 10^6^ cells per well in a 12- or 6-well plate, respectively, in complete DMEM/F12. Prior to seeding the wells had been coated either with 0.1% (m/V) poly-L-ornithine (PLO, Gibco) in PBS for 2 h at room temperature or 2 µg/mL collagen IV for 2 h at 37 °C. Microglia were allowed to adhere to the wells for 30 min before the medium was replaced. The brief initial exposure to complete DMEM/F12 containing 10% FBS during the adhesion phase was identical in all experimental conditions and is taken into account in the interpretation of differences between serum-free and serum-containing cultures. Different medium supplements were used alone or in combination for the cultivation of microglia isolated using the shaking method: no FBS or 10% FBS; 1.5 µg/mL cholesterol (from sheep, Avanti Polar Lipids Inc.); 100 ng/mL human TGF-β1 (Peprotech, 100–21); 100 ng/mL human IL-34 (Peprotech, 200–34); 100 ng/mL human CX3CL1 (Peprotech, 300–31); 10–500 µg/mL urea (Carl Roth); 0.1–10 µg/mL sodium selenite (Merck); 0.1–1x insulin-transferrin-selenium solution (Life Technologies). Recommended combined optimized conditions are shown in Table 2. The cells were incubated at 37 °C with 5% CO_2_ for 1–7 days.

**Table 2:** Combined optimized culture conditions for neonatal primary microglia culture in serum-free DMEM/F12 recommended by the authors. Gentamicin (50 µg/mL) is also included.

Supplement	Concentration
Collagen IV (vessel coating)	2 µg/mL (leave for 2 h at 37 °C, wash 1x with PBS)
hTGF-β1	100 ng/mL
hIL-34	100 ng/mL
hCX3CL1	100 ng/mL
Cholesterol	1.5 µg/mL
insulin-transferrin-selenium solution	0.1x

### Immunocytochemistry

Isolated microglia were seeded at a density of 5 × 10^4^ cells per well on chamber slides (Corning Inc.) and cultured for 24 h. Medium was removed, slides were rinsed with PBS, and cells were fixed in 4% paraformaldehyde (Thermo Fisher Scientific) for 15 min at room temperature. Then, slides were washed three times with 50 mM ammonium chloride in PBS, permeabilized with 0.1% saponin (Sigma-Aldrich) in PBS for 10 min, and blocked for 30 min in 3% goat serum (Invitrogen)/0.1% saponin in PBS. Primary antibodies were applied, slides were sealed with Parafilm, and incubated overnight at 4°C. The next day, chambers were washed twice with 0.1% saponin in PBS, re-blocked for 10 min in 3% goat serum/0.1% saponin in PBS, and incubated with secondary antibodies (200 µL per chamber) for 1 h at room temperature. Slides were washed twice with 0.1% saponin in PBS and twice with PBS. Nuclei were counterstained with DAPI (1 µg/mL, Life Technologies) for 2 min at room temperature in the dark, followed by two PBS washes. Chambers were removed, slides air-dried, and mounted with Citifluor AF1 (Citifluor Ltd). Mounted slides were dried overnight at 4°C in the dark and imaged using a Zeiss Axiovert S100. The following antibodies and dilutions were used: anti-CD11b (BioRad AbD Serotec, MCA711, 1:50) anti-GFAP (Dako Agilent, Z0334, 1:500), anti-Iba1 (Wako Chemicals, 019-19741, 1:200), anti-NeuN (Abcam, ab177487, 1:200), Cy2-goat-anti-rabbit (Dianova, 111-225-144, 1:50), Cy2-goat-anti-rat (Dianova, 112-225-167, 1:50).

### RNA isolation

Total RNA was isolated from cell pellets or lysates using the Nucleospin RNA XS isolation kit (Machery-Nagel) with slight adaptations to the manufacturer’s protocol: 25 µL carrier RNA working solution was added to the lysate instead of 5 µL; 500 µL of 70% ethanol was added to the filtered lysate instead of 100 µL; RNA elution was performed using 12 µL of RNAse-free water instead of 10 µL; and the eluate was reloaded onto the column again to increase the yield. RNA concentration and quality were determined using a Nanodrop 2000 (Thermo Scientific). RNA samples were stored at −80 °C until further use.

### Complementary DNA (cDNA) Generation and RT-qPCR

Transcription of isolated RNA into cDNA was achieved by mixing 200 ng total RNA with 1 µL of 100 µM random hexanucleotide primers (Roche Diagnostics GmbH), 1 µL of 10 mM dNTPs (Thermo Fisher Scientific), and then filling the mixture to 14 µL with nuclease-free water, followed by incubation for 5 min at 65 °C. Next, 4 µL of 5x first-strand buffer (Thermo Fisher Scientific), 2 µL of a 100 mM DTT solution (Thermo Fisher Scientific), 0.5 µL of SuperScript II reverse transcriptase (Life Technologies), and 1.5 µL of nuclease-free water were added. The preparation was incubated for 10 min at 25 °C, followed by 50 min at 42 °C and finally 15 min at 70 °C.

Quantification of gene expression of microglia-specific genes (*Cx3cr1*,* Tgfbr1*,* Tmem119*,* P2ry12*,* Hexb*,* Fcrl2*,* Olfml3*) by reverse transcription quantitative PCR (RT-qPCR) was performed using SYBR Green PCR Mastermix (Qiagen). Each reaction contained 1 µL of cDNA, 500 nM of forward and reverse primer, 7.5 µL SYBR Green Mastermix and water up to 15 µL. Amplicon length was limited to 240 base pairs. The melting temperatures of the primers were set to be in the range of 60–63 °C. Used primer sequences are listed Table 3. Thermal cycling and detection were performed in a Rotor Gene RG-3000 cycler (Corbett Research/Qiagen). After an initial denaturation step at 95 °C for 5 min, amplification was performed by 40 cycles with denaturation at 95 °C for 5 s and primer annealing and extension at 60 °C for 15 s. *Gapdh* or *Ywhaz* were used as reference genes for normalization and quantification was performed according to the 2^−ΔΔCt^ method^51^. Results are presented as 2^−ΔΔCt^ (fold change) on log 2 axis. Statistical tests are performed on ΔCt values (Ct _gene of interest_ – Ct _reference gene_), since fold changes are multiplicative, skewed, and often heteroscedastic^51^. We are aware that the expression of housekeeping genes such as *Gapdh* and *Ywhaz* can be modulated by microglial activation state and culture conditions; the use of these genes as single references therefore represents a limitation of the normalization strategy and is taken into account when interpreting the RT-qPCR data.

**Table 3:** Primers used for RT-qPCR.

Gene	Forward primer	Reverse primer	Amplicon length [bp]
*Cx3cr1*	CTCACCATGTCCACCTCCTT	CGAGGACCACCAACAGATTT	172
*Hexb*	TTGATACCCCTGGCCATAC	TCATCTCCTCCCAAGTGG	199
*Olfml3*	TGGGGCCAGCAGAGAAGA	TCCAGGAGGCCTTCGAGC	194
*P2ry12*	AGCCGGGCCTCTGAGAAC	TTGGCCTCCTGTTGGTGA	239
*Tgfbr1*	TCATTTCAGAGGGCACCAC	TCTGCCTCTCGGAACCAT	232
*Tmem119*	TGTGAAGGGTGGGTCGGA	ACCAGTTGCTCCCTGGGA	214
*Fcrl2*	TCGTTGAAGGGGAGCCCC	TGCACTCTGGATCAGGCC	215
*Gapdh*	ACTCCACTCACGGCAAATTC	TCTCCATGGTGGTGAAGACA	171
*Ywhaz*	TCTTGTCACCAACCATTCCA	CATTTAGGGCAGGACTTCCA	192

### Microarray and RNA sequencing

DNA microarray analysis was performed by Oaklabs (Henningsdorf, Germany). Sample hybridization was performed on Agilent ArrayXS_Mouse_V1 microarrays. Quantile normalized data were used for all further calculations. Cluster analysis was performed with Genesis^52^. Paired end RNA sequencing was performed essentially as described^53^ by Biomarker Technologies (Münster, Germany). Reads were mapped to the murine genome version mm39 using HISAT2. Quantification of mapped reads was performed using featureCounts. Quality control was performed with fastQC. All procedures were performed with Galaxy (usegalaxy.eu;^54^). Volcano plots were prepared with VolcaNoseR (https://huygens.science.uva.nl/VolcaNoseR/;^55^). Microglia marker genes^4^ were filtered from count tables by using Microsoft Excel. For ex vivo microglia control samples, cells were isolated and processed for RNA extraction as described under “Brain Dissection and Dissociation” and “Microglia isolation”, i.e., using enzymatic digestion in the presence of FBS and without transcriptional inhibitors. The resulting transcriptomes therefore reflect the state of microglia after this isolation procedure rather than an undisturbed in vivo state, which is considered when interpreting comparisons between cultured and ex vivo microglia.

### Statistical analyses

Sample numbers (n) are defined as independent biological replicates and are indicated in figure legends. Statistical evaluations were performed using GraphPad Prism Software. Statistics on microarray and RT-qPCR data were performed using ΔCt values and based on α = 5%. Using one way analysis of variance (ANOVA) followed by post-hoc tests (Dunnet’s or Tukey’s), α depicts the family-wise error rate. However, to facilitate understanding of the data, significance levels are displayed in graphs showing 2^−ΔΔCt^ values, not ΔCt values.

## Supplementary Information

Below is the link to the electronic supplementary material.

Supplementary Material 1

## Data Availability

The datasets generated and/or analyzed during the current study are available in the Gene Expression Omnibus (GEO) repository. The RNA-seq data are available under the following accession number: GSE316250. The microarray data are available under the following accession number: GSE316252.

## References

[1] Prinz, M., Jung, S. & Priller, J. Microglia biology: One century of evolving concepts. *Cell* **179**, 292–311. 10.1016/j.cell.2019.08.053 (2019). 31585077 10.1016/j.cell.2019.08.053

[2] Li, Q. & Barres, B. A. Microglia and macrophages in brain homeostasis and disease. *Nat. Rev. Immunol.* **18**, 225–242. 10.1038/nri.2017.125 (2018). 29151590 10.1038/nri.2017.125

[3] Stevens, B. et al. The classical complement cascade mediates CNS synapse elimination. *Cell* **131**, 1164–1178. 10.1016/j.cell.2007.10.036 (2007). 18083105 10.1016/j.cell.2007.10.036

[4] Butovsky, O. et al. Identification of a unique TGF-β-dependent molecular and functional signature in microglia. *Nat. Neurosci.* **17**, 131–143. 10.1038/nn.3599 (2014). 24316888 10.1038/nn.3599 PMC4066672

[5] Bennett, M. L. et al. New tools for studying microglia in the mouse and human CNS. *Proc. Natl. Acad. Sci. U.S.A.* **113**, E1738–E1746. 10.1073/pnas.1525528113 (2016). 26884166 10.1073/pnas.1525528113 PMC4812770

[6] Pesti, I., Légrádi, Á. & Farkas, E. Primary microglia cell cultures in translational research: Strengths and limitations. *J. Biotechnol.* **386**, 10–18. 10.1016/j.jbiotec.2024.03.005 (2024). 38519034 10.1016/j.jbiotec.2024.03.005

[7] Gosselin, D. et al. An environment-dependent transcriptional network specifies human microglia identity. *Sci. (New York N Y)*. **356**, eaal3222. 10.1126/science.aal3222 (2017). 10.1126/science.aal3222 PMC5858585 28546318

[8] Bohlen, C. J. et al. Diverse requirements for microglial survival, specification, and function revealed by defined-medium cultures. *Neuron* **94**, 759–773. 10.1016/j.neuron.2017.04.043 (2017). 28521131 10.1016/j.neuron.2017.04.043 PMC5523817

[9] Cadiz, M. P. et al. Culture shock: Microglial heterogeneity, activation, and disrupted single-cell microglial networks in vitro. *Mol. Neurodegen*. **17**, 26 10.1186/s13024-022-00531-1 (2022). 10.1186/s13024-022-00531-1 PMC8962153 35346293

[10] Abud, E. M. et al. iPSC-derived human microglia-like cells to study neurological diseases. *Neuron* **94**, 278–293e9. 10.1016/j.neuron.2017.03.042 (2017). 28426964 10.1016/j.neuron.2017.03.042 PMC5482419

[11] Kierdorf, K. & Prinz, M. Microglia in steady state. *J. Clin. Investig.* **127**, 3201–3209. 10.1172/JCI90602 (2017). 28714861 10.1172/JCI90602 PMC5669563

[12] Wolf, Y., Yona, S., Kim, K. W. & Jung, S. Microglia, seen from the CX3CR1 angle. *Front. Cell. Neurosci.* **7**, 26. 10.3389/fncel.2013.00026 (2013). 23507975 10.3389/fncel.2013.00026 PMC3600435

[13] Zöller, T. et al. Silencing of TGFβ signalling in microglia results in impaired homeostasis. *Nat. Commun.* **9**, 4011. 10.1038/s41467-018-06224-y (2018). 30275444 10.1038/s41467-018-06224-y PMC6167353

[14] Wang, Y. et al. IL-34 is a tissue-restricted ligand of CSF1R required for the development of Langerhans cells and microglia. *Nat. Immunol.* **13**, 753–760. 10.1038/ni.2360 (2012). 22729249 10.1038/ni.2360 PMC3941469

[15] Nayak, D., Roth, T. L. & McGavern, D. B. Microglia development and function. *Annu. Rev. Immunol.* **32**, 367–402. 10.1146/annurev-immunol-032713-120240 (2014). 24471431 10.1146/annurev-immunol-032713-120240 PMC5001846

[16] Elmore, M. R. P. et al. Colony-stimulating factor 1 receptor signaling is necessary for microglia viability, unmasking a microglia progenitor cell in the adult brain. *Neuron* **82**, 380–397. 10.1016/j.neuron.2014.02.040 (2014). 24742461 10.1016/j.neuron.2014.02.040 PMC4161285

[17] Mizuno, T. et al. Interleukin-34 selectively enhances the neuroprotective effects of microglia to attenuate oligomeric amyloid-β neurotoxicity. *Am. J. Pathol.* **179**, 2016–2027. 10.1016/j.ajpath.2011.06.011 (2011). 21872563 10.1016/j.ajpath.2011.06.011 PMC3181379

[18] Barger, S. W. & Basile, A. S. Activation of microglia by secreted amyloid precursor protein evokes release of glutamate by cystine exchange and attenuates synaptic function. *J. Neurochem.* **76**, 846–854. 10.1046/j.1471-4159.2001.00075.x (2001). 11158256 10.1046/j.1471-4159.2001.00075.x

[19] Li, L., Sun, B., Harris, O. A. & Luo, J. TGF-β signaling in microglia: A key regulator of development, homeostasis and reactivity. *Biomedicines* **12**, 2468. 10.3390/biomedicines12112468 (2024). 39595034 10.3390/biomedicines12112468 PMC11592028

[20] Kimura, K. et al. Immune checkpoint TIM-3 regulates microglia and Alzheimer’s disease. *Nature* **641**, 718–731. 10.1038/s41586-025-08852-z (2025). 40205047 10.1038/s41586-025-08852-z PMC12079183

[21] Zhu, Q., Wang, Y., Liu, Y., Yang, X. & Shuai, Z. Prostate transmembrane androgen inducible protein 1 (PMEPA1): Regulation and clinical implications. *Front. Oncol.* **13**, 1298660. 10.3389/fonc.2023.1298660 (2023). 38173834 10.3389/fonc.2023.1298660 PMC10761476

[22] Krawczyk, M. C., Godoy, M., Vander, P., Zhang, A. J. & Zhang, Y. Loss of Serpin E2 alters antimicrobial gene expression by microglia but not astrocytes. *Neurosci. Lett.* **811**, 137354. 10.1016/j.neulet.2023.137354 (2023). 37348749 10.1016/j.neulet.2023.137354 PMC11473033

[23] Rotterman, T. M. et al. Modulation of central synapse remodeling after remote peripheral injuries by the CCL2-CCR2 axis and microglia. *Cell. Rep.* **43**, 113776. 10.1016/j.celrep.2024.113776 (2024). 38367237 10.1016/j.celrep.2024.113776 PMC10947500

[24] Yi, L. et al. NOD2 promotes sepsis-induced neuroinflammation by increasing brain endoplasmic reticulum stress mediated by LACC1. *Free Radic. Biol. Med.* **235**, 280–293. 10.1016/j.freeradbiomed.2025.05.002 (2025). 40335000 10.1016/j.freeradbiomed.2025.05.002

[25] Yu, S. et al. Fascin-1 is highly expressed specifically in microglia after spinal cord injury and regulates microglial migration. *Front. Pharmacol.* **12**, 729524. 10.3389/fphar.2021.729524 (2021). 34646136 10.3389/fphar.2021.729524 PMC8502808

[26] Jiang, J. et al. Prolactin deficiency drives diabetes-associated cognitive dysfunction by inducing microglia-mediated synaptic loss. *J. Neuroinflamm*. **21**, 295. 10.1186/s12974-024-03289-z (2024). 10.1186/s12974-024-03289-z PMC11566644 39543619

[27] Lloyd, A. F. et al. Deep proteomic analysis of microglia reveals fundamental biological differences between model systems. *Cell. Rep.* **43**, 114908. 10.1016/j.celrep.2024.114908 (2024). 39460937 10.1016/j.celrep.2024.114908

[28] Montilla, A., Zabala, A., Matute, C. & Domercq, M. Functional and metabolic characterization of microglia culture in a defined medium. *Front. Cell. Neurosci.* **14**, 22. 10.3389/fncel.2020.00022 (2020). 32116565 10.3389/fncel.2020.00022 PMC7025516

[29] Ingold, I. et al. Selenium utilization by GPX4 is required to prevent hydroperoxide-induced ferroptosis. *Cell* **172**, 409–422e21. 10.1016/j.cell.2017.11.048 (2018). 29290465 10.1016/j.cell.2017.11.048

[30] Zhang, Z. H. & Song, G. L. Roles of selenoproteins in brain function and the potential mechanism of selenium in Alzheimer’s disease. *Front. NeuroSci.* **15**, 646518. 10.3389/fnins.2021.646518 (2021). 33762907 10.3389/fnins.2021.646518 PMC7982578

[31] Darwish, R. et al. The role of hypothalamic microglia in the onset of insulin resistance and type 2 diabetes: A neuro-immune perspective. *Int. J. Mol. Sci.* **25**, 13169. 10.3390/ijms252313169 (2024). 39684879 10.3390/ijms252313169 PMC11642714

[32] Chen, W., Liu, X., Muñoz, V. R. & Kahn, C. R. Loss of insulin signaling in microglia impairs cellular uptake of Aβ and neuroinflammatory response exacerbating AD-like neuropathology. *Proc. Natl. Acad. Sci. U.S.A.* **122**, e2501527122. 10.1073/pnas.2501527122 (2025). 40388612 10.1073/pnas.2501527122 PMC12130885

[33] Galy, B., Conrad, M. & Muckenthaler, M. Mechanisms controlling cellular and systemic iron homeostasis. *Nat. Rev. Mol. Cell Biol.* **25**, 133–155. 10.1038/s41580-023-00648-1 (2024). 37783783 10.1038/s41580-023-00648-1

[34] Kulaszyńska, M., Kwiatkowski, S. & Skonieczna-Żydecka, K. The iron metabolism with a specific focus on the functioning of the nervous system. *Biomedicines* **12** (3), 595. 10.3390/biomedicines12030595 (2024). 38540208 10.3390/biomedicines12030595 PMC10968467

[35] Muñoz-Garcia, J. et al. The twin cytokines interleukin-34 and CSF-1: Masterful conductors of macrophage homeostasis. *Theranostics* **11**, 1568–1593. 10.7150/thno.50683 (2021). 33408768 10.7150/thno.50683 PMC7778581

[36] Huang, S. et al. Mutation in the rat interleukin 34 gene impacts macrophage development, homeostasis, and inflammation. *Life Sci. Alliance*. **8**, 9. 10.26508/lsa.202503264 (2025). 10.26508/lsa.202503264 PMC12177736 40533345

[37] Limatola, C. & Ransohoff, R. M. Modulating neurotoxicity through CX3CL1/CX3CR1 signaling. *Front. Cell. Neurosci.* **8**, 229. 10.3389/fncel.2014.00229 (2014). 25152714 10.3389/fncel.2014.00229 PMC4126442

[38] Paolicelli, R. C. et al. Synaptic pruning by microglia is necessary for normal brain development. *Sci. (New York N Y)*. **333**, 1456–1458. 10.1126/science.1202529 (2011). 10.1126/science.1202529 21778362

[39] McQuade, A. et al. Development and validation of a simplified method to generate human microglia from pluripotent stem cells. *Mol. Neurodegener.* **13**, 67. 10.1186/s13024-018-0297-x (2018). 10.1186/s13024-018-0297-x PMC6303871 30577865

[40] Haenseler, W. et al. A highly efficient human pluripotent stem cell microglia model displays a neuronal-co-culture-specific expression profile and inflammatory response. *Stem cell. Rep.* **8**, 1727–1742. 10.1016/j.stemcr.2017.05.017 (2017). 10.1016/j.stemcr.2017.05.017 PMC5470330 28591653

[41] Wareham, L. K. & Calkins, D. J. Making tracks: Microglia and the extracellular matrix. *Mol. Neurodegener*. **20**, 101. 10.1186/s13024-025-00898-x (2025). 10.1186/s13024-025-00898-x PMC12481997 41024112

[42] Ulland, T. K. et al. TREM2 maintains microglial metabolic fitness in Alzheimer’s disease. *Cell* **170**, 649–663e13. 10.1016/j.cell.2017.07.023 (2017). 28802038 10.1016/j.cell.2017.07.023 PMC5573224

[43] Nugent, A. A. et al. TREM2 regulates microglial cholesterol metabolism upon chronic phagocytic challenge. *Neuron* **105**, 837–854e9. 10.1016/j.neuron.2019.12.007 (2020). 31902528 10.1016/j.neuron.2019.12.007

[44] Baxter, P. S. et al. Microglial identity and inflammatory responses are controlled by the combined effects of neurons and astrocytes. *Cell. Rep.* **34**, 108882. 10.1016/j.celrep.2021.108882 (2021). 33761343 10.1016/j.celrep.2021.108882 PMC7994374

[45] Badley, J. R., Bhusal, A. & Lein, P. J. A primary rat neuron-astrocyte-microglia tri-culture model for studying mechanisms of neurotoxicity. *Front. Toxicol.* **6**, 1523387. 10.3389/ftox.2024.1523387 (2024). 39867128 10.3389/ftox.2024.1523387 PMC11759268

[46] Luchena, C. et al. A Neuron, microglia, and astrocyte triple co-culture model to study Alzheimer’s disease. *Front. Aging Neurosci.* **14**, 844534. 10.3389/fnagi.2022.844534 (2022). 35493929 10.3389/fnagi.2022.844534 PMC9048896

[47] Tewari, M. et al. Physiology of cultured human microglia maintained in a defined culture medium. *ImmunoHorizons* **5**, 257–272. 10.4049/immunohorizons.2000101 (2021). 33931497 10.4049/immunohorizons.2000101 PMC9190148

[48] Dorion, M. F. et al. Systematic comparison of culture media uncovers phenotypic shift of primary human microglia defined by reduced reliance to CSF1R signaling. *Glia* **71**, 1278–1293. 10.1002/glia.24338 (2023). 36680780 10.1002/glia.24338

[49] Pösel, C., Möller, K., Boltze, J., Wagner, D. C. & Weise, G. Isolation and flow cytometric analysis of immune cells from the ischemic mouse brain. *J. Visual. Exp.: JoVE*. 10.3791/53658 (2016). 10.3791/53658 PMC4828148 26967380

[50] Sanford, K. K., Likely, G. D. & Earle, W. R. The growth in vitro of single isolated tissue cells. *J. Natl. Cancer Inst.* **9**, 229–246 (1948). 18105872

[51] Livak, K. J. & Schmittgen, T. D. Analysis of relative gene expression data using real-time quantitative PCR and the 2(-Delta Delta C(T)) Method. *Methods (San Diego Calif)*. **25**, 402–408. 10.1006/meth.2001.1262 (2001). 11846609 10.1006/meth.2001.1262

[52] Sturn, A., Quackenbush, J. & Trajanoski, Z. Genesis: Cluster analysis of microarray data. *Bioinf. (Oxford England)*. **18**, 207–208. 10.1093/bioinformatics/18.1.207 (2002). 10.1093/bioinformatics/18.1.207 11836235

[53] Engel, K. et al. Identification of differentially expressed human endogenous retrovirus families in human leukemia and lymphoma cell lines and stem cells. *Front. Oncol.* **11**, 637981. 10.3389/fonc.2021.637981 (2021). 33996550 10.3389/fonc.2021.637981 PMC8117144

[54] The Galaxy Community. The Galaxy platform for accessible, reproducible, and collaborative data analyses: 2024 update. *Nucleic Acids Res.* **52**, W83–W94. 10.1093/nar/gkae410 (2024). 38769056 10.1093/nar/gkae410 PMC11223835

[55] Goedhart, J. & Luijsterburg, M. S. VolcaNoseR is a web app for creating, exploring, labeling and sharing volcano plots. *Sci. Rep.* **10**, 20560. 10.1038/s41598-020-76603-3 (2020). 33239692 10.1038/s41598-020-76603-3 PMC7689420

