# Supplementary material for: Reconstituting central nervous system niche cues partially restores homeostatic-like features in cultured murine primary microglia
Source: Sci Rep. 2026 Jul 7;16:20998. doi: 10.1038/s41598-026-61142-0 (PMC13341787; doi:10.1038/s41598-026-61142-0)
Supplement: Supplementary file 1 — Supplementary Material 1 [file 41598_2026_61142_MOESM1_ESM.docx]

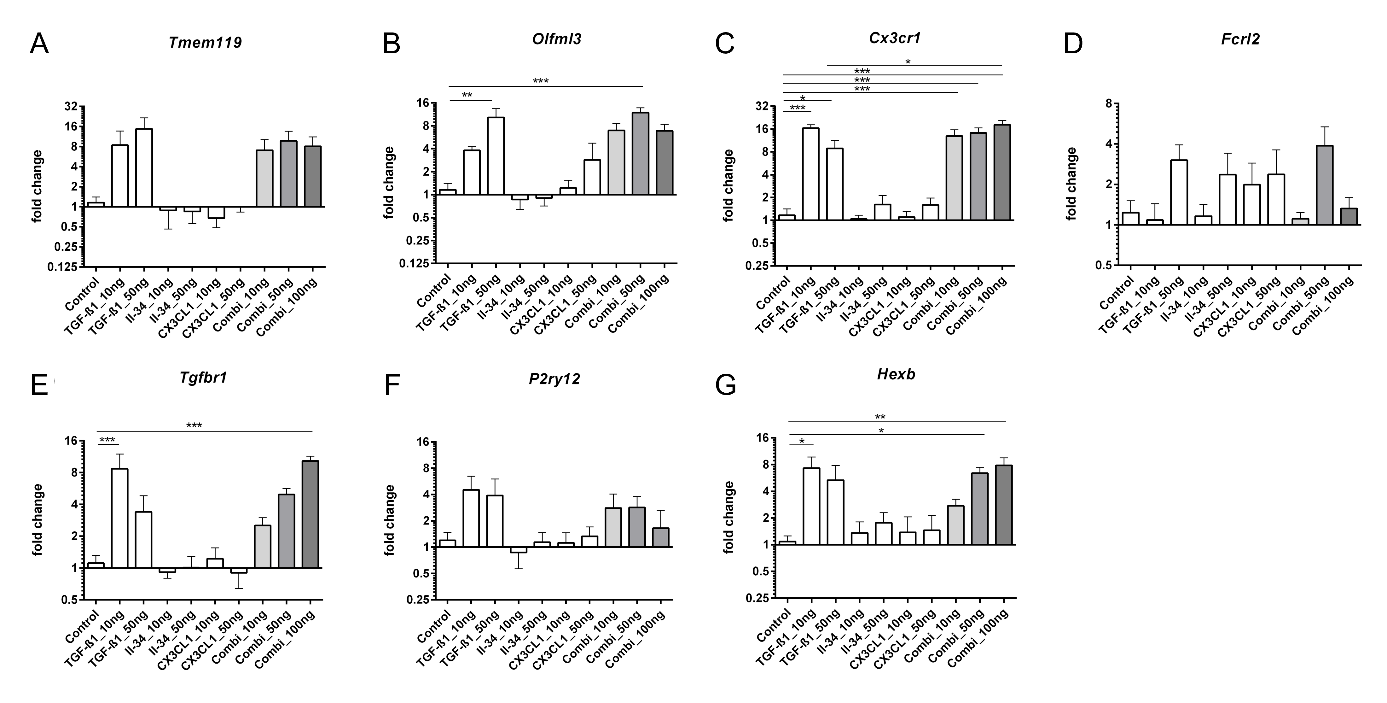


***Supplementary Figure 1****. RT-qPCR analysis of cytokine effects on seven signature genes in neonatal murine primary microglia. (A-G) Microglia were isolated from neonatal C57BL/6N mice by shaking method and cultured for 24 h in 12-well plates in DMEM/F12 + 10% FBS + 50 µg/mL gentamicin (control) supplemented with three different cytokines (TGF-β1, IL-34, CX3CL1) alone or in combination as indicated. Data are presented as fold change (2^-ΔΔCt^) normalized to Ywhaz and expressed relative to the mean of the control; mean ± SEM. n=3-9. Statistics: One‑way ANOVA with Tukey’s multiple comparison test, only p-values for comparisons vs. control are shown. A–G, individual genes as labeled. Significance: * P<0.05; ** P<0.01; *** P<0.001.*


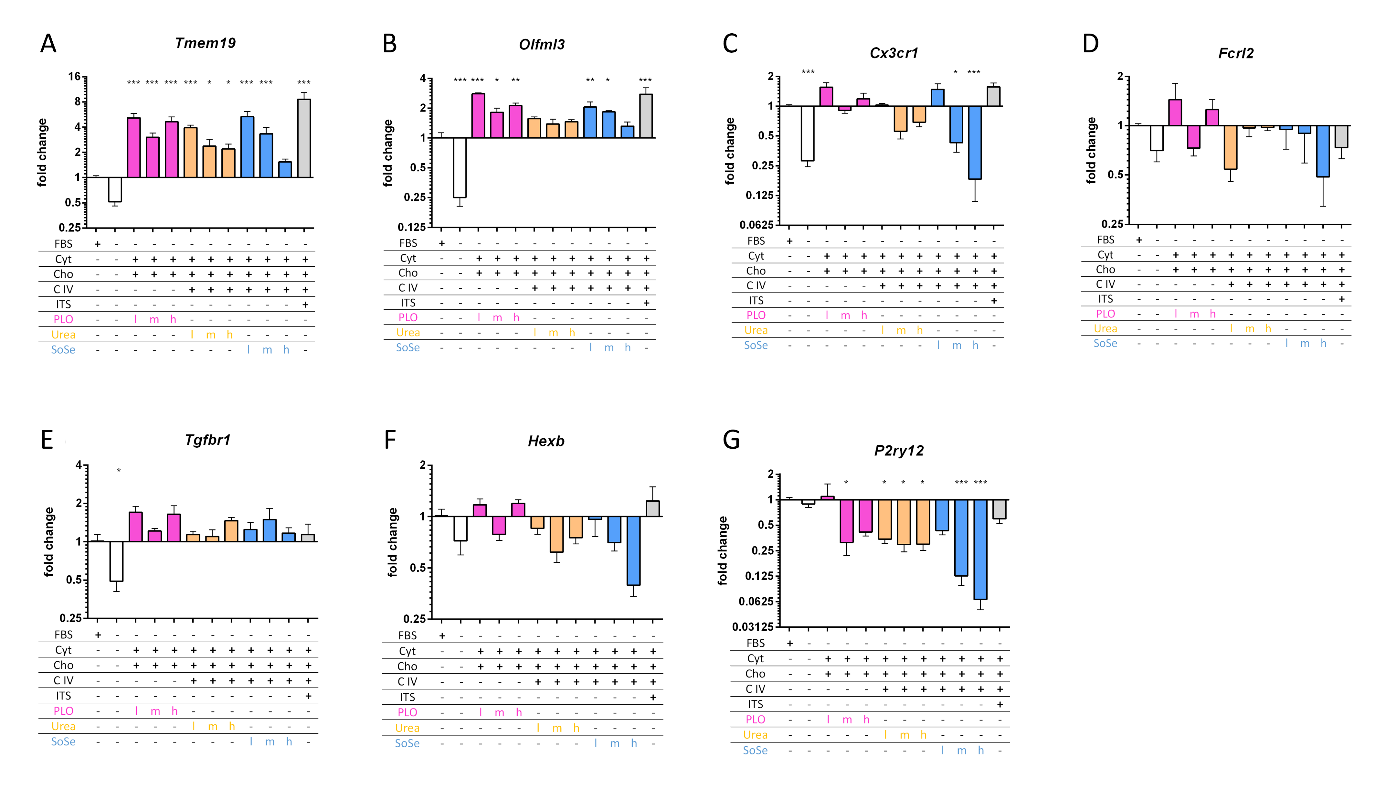


***Supplementary Figure 2****. RT-qPCR analysis of cytokine effects on seven signature genes in neonatal murine primary microglia. (A-G) Microglia were isolated from neonatal C57BL/6N mice by shaking method and cultured for 24 h in 12-well plates in DMEM/F12 + 50 µg/mL gentamicin (control) and varying medium supplements: fetal bovine serum (FBS, 10%); TGF-β1, IL-34, CX3CL1 (Cyt, 100 ng/mL each); cholesterol (Cho, 1.5 µg/mL); insulin-transferrin-selenium solution (ITS, 0.1x); different concentrations of urea (low (l): 10 µg/mL, medium (m): 50 µg/mL, high (h): 500 µg/mL); different concentrations of sodium selenite (SoSe, low (l): 0.1 µg/mL, medium (m): 1 µg/mL, high (h): 10 µg/mL). Well plates were coated with 2 µg/mL collagen-IV (C IV) or different concentrations of poly-l-ornithine (PLO, low (l): 0.001%, medium (m): 0.01%, high (h): 0.1%) prior to seeding cells as indicated. Data are presented as fold change (2^-ΔΔCt^) normalized to Gapdh and expressed relative to the mean of the control; mean ± SEM. n=3-9. Statistics: One‑way ANOVA with Tukey’s multiple comparison test, only p-values for comparisons vs. control are shown. A–G, individual genes as labeled. Significance: * P<0.05; ** P<0.01; *** P<0.001.*
